# Diversity and altitudinal distribution of phlebotomine sand flies (Diptera: Psychodidae) in visceral leishmaniasis endemic areas of northwest Ethiopia

**DOI:** 10.1016/j.actatropica.2017.07.008

**Published:** 2017-12

**Authors:** Solomon Yared, Araya Gebresilassie, Essayas Akililu, Kebede Deribe, Meshesha Balkew, Alon Warburg, Asrat Hailu, Teshome Gebre-Michael

**Affiliations:** a Department of Biology, College of Natural and Computational Science, Jigjiga University, Jigjiga, Ethiopia; b Department of Biology, Mada Walabu University, Bale-Robe, Ethiopia; c Wellcome Trust Brighton and Sussex Centre for Global Health Research, Brighton and Sussex Medical School, Brighton, United Kingdom. School of Public Health, Addis Ababa University, Addis Ababa, Ethiopia; d Aklilu Lemma Institute of Pathobiology, Addis Ababa University, Addis Ababa, Ethiopia; e Department of Microbiology and Molecular Genetics, The Institute of Medical Research Israel-Canada, The Kuvin Center for the Study of Infectious and Tropical Diseases, Faculty of Medicine, The Hebrew University, Hadassah Medical School, Jerusalem, Israel; f Department of Microbiology, Immunology and Parasitology, Faculty of Medicine, Addis Ababa University, Addis Ababa, Ethiopia

**Keywords:** Shannon-Weiner, Species richness, *Phlebotomus*, Sergentomyia, Leishmaniasis, Altitudinal transect

## Abstract

**Background:**

The Leishmaniases are caused by the protozoan parasites of the genus *Leishmania* and are transmitted to humans by the bite of infected female phlebotomine sand flies. Both visceral and cutaneous leishmaniases are widely distributed in different parts of Ethiopia. The aim of this study was to determine the diversity and altitudinal distribution of phlebotomine sand flies from Kafta Humera to Gondar town in northwest Ethiopia.

**Methods:**

Seven localities were selected with distinct altitudinal variations between 550 m above sea level (m a.s.l) and 2300 m a.s.l. In each locality, sand flies were collected using standard CDC light traps and sticky traps during the active sand fly season from December 2012 to May 2013. Shannon-Weiner species diversity index and Jaccard’s coefficient were used to estimate species diversity and similarity between altitudes and localities, respectively.

**Results:**

A total of 89,044 sand flies (41,798 males and 47, 246 females) were collected from the seven localities/towns throughout the study period. Twenty-two species belonging to 11 species in the genus *Phlebotomus* and 11 species in the genus *Sergentomyia* were documented. Of these, *Sergentomyia clydei* (25.87%), *S. schwetzi* (25.21%), *S. africana* (24.65%), *S. bedfordi* (8.89%), *Phlebotomus orientalis* (6.43%), and *S. antennata* (4.8%) were the most prevalent species. The remaining 10 *Phlebotomus* species and six *Sergentomyia* were less frequent catches. In CDC light trap and sticky trap, higher species diversity and richness for both male and female sand flies was observed at low altitude ranging from 550 to 699 m a.s.l in Adebay village in Kafta Humera district whereas low species richness and high evenness of both sexes were also observed in an altitude 1950–2300 m a.s.l.

**Conclusion:**

The results revealed that the presence of leishmaniasis vectors such as *P. orientalis, P. longipes, P. papatasi,* and *P. duboscqi* in different altitudes in northwest Ethiopia. *P. orientalis* a vector of *L. donovani*, occurred between altitude 500–1100 m a.s.l, the area could be at high risk of VL. *P. longipes* a vector of *L. aethiopica*, was recorded in the highland area in Tikil-Dingay and Gondar town, implicating the possibility of CL transmission. Hence, further investigation into vector competence in relation to leishmaniasis (VL and CL) in the region is very vital.

## 1 Introduction

Phlebotomine sand flies are involved in the transmission of leishmaniases, sand fly fever and bartonellosis in the tropical and subtropical regions of the Old and New Worlds. In the Old World, phlebotomine sand flies belong to three genera: *Phlebotomus*, *Sergentomyia* and *Chinius*. Only species occurring in the genus *Phlebotomus* are responsible in the transmission of leishmaniasis and sand fly fever in the Old World (Lane, 1993, WHO, 2010).

Leishmaniasis exist in two main clinical forms: visceral leishmaniasis (VL) and cutaneous leishmaniasis (CL) which both occur in different parts of Ethiopia. Cutaneous leishmaniasis is caused by three different *Leishmania* species: *L. aethiopica*, *L. major* and *L tropica*, however, CL due to *L. aethiopica* is by far the most widespread and important disease in Ethiopia (Hailu et al., 2006). The estimated annual incidence of CL is about 20,000–50,000 cases with a population of about 28 million at risk (Alvar et al., 2012, Tsegaw et al., 2013). It mainly occurs between 1400 and 2900 m a.s.l (Wilkins, 1972, Ashford et al., 1973, Hailu et al., 2006). In contrast, VL is mainly found in the peripheral low land areas of the south, southwest, north and northwest Ethiopia (1500 m a.s.l) (Hailu et al., 2006) but has recently been emerged in highland areas ranging from 1800 to 2000 m a.s.l (Alvar et al., 2007, Herrero et al., 2009). The estimated annual incidence of VL is between 3700 and 7400 cases (Alvar et al., 2012, Deribe et al., 2012), and the population at risk is about 3.3 million in Ethiopia (Tsegaw et al., 2013).

In Ethiopia, at least 22 species of *Phlebotomus* have been documented, of which eight species have been proved or suspected as vectors of CL and VL, *P. martini*, *P. orientalis* and *P. celiae* are vectors of VL due to *L. donovani* while *P. longipes*, *P. pedifer* are vectors of *L. aethiopica* (CL), *P. duboscqi* is vector of *L. major* (CL) and *L. tropica* (CL) transmission is associated with *P. sergenti* and *P. saevus* (Gebre-Michael et al., 2004a, Hailu et al., 2006, WHO, 2010). With regards to the distribution of vectors of VL, they are widely spread in different ecological settings. Two species of the subgenus *Synphlebotomus P. martini* and *P. celiae* and another related species *P. vansomernae* (its role yet unknown) are associated with *Macrotermes* termite mounds (Minter, 1964, Marlet et al., 2003). These are generally found in the southern parts of the country, but *P. martini* have been found as far north as Thatay Adiabo district in Tigray Region (Gebresilassie et al., 2015). *Phlebotomus orientalis* which is the most likely VL vector in north and northwest Ethiopia (Hailu et al., 1995, Gebre-Michael et al., 2010) is mostly distributed in areas where there are *Acacia-Balanites* vegetation and cracking black cotton clay soil (verstisol) as in Sudan and South Sudan (Hoogstraal and Heyneman, 1969, Elnaiem et al., 1999). In Ethiopia, *P. orientalis* has an extensive geographical distribution much more than the distribution of the disease (Gebre-Michael et al., 2004b).

Phlebotomine sand flies are widely distributed along different elevation gradients. The density of sand flies at higher and lower altitudes is dependent on different environmental and climatic conditions. The distribution and abundance of sand fly vectors and human and/or reservoir hosts are affected by various physical factors (temperature, rain fall, humidity, altitude, latitude, surface water and wind) and biotic factors (vegetation, host species, predators, competitors, parasites and human interventions) (Lane, 1993, Rohr et al., 2011). All of these factors also affect the spatial and temporal distribution of vectors and reservoirs, which in turn affect the epidemiology and dynamics of pathogen transmission to the human population (Rohr et al., 2011). Knowledge on diversity and altitudinal distribution of sand flies are very vital to predict the impact of environmental modification, the increasing seasonal labourer migration (from non-endemic to endemic areas and the vice versa) and climate change on the dynamics of vector population. Currently, cases of VL and CL have been reported in different parts of the country, showing that both diseases are spreading in previously non-endemic areas. A few years ago, few cases of VL were diagnosed in Gondar town (2300 m a.s.l) in children who have never been out of the town (Prof A. Hailu). VL cases have also been detected in some districts between Kafta Humera and towns near Gondar towns (e.g. Dansha, Sorkoa, Sanja) (Humera Hospital, unpublished), 1000–2000 cases occur annually in Kafta Humera-Metema plains (Ngure et al., 2009). In Kafta Humera, a case-control study showed that daily individual activities around home and farm fields during night times and poor housing conditions are important for VL transmission (Yared et al., 2014). The spreading of VL and CL in the country may be due to environmental change, demographic, host and human activity factors (Desjex, 2001). As well, the distribution and abundance of the sand fly vector are also enhancing the transmission of the disease (Desjex, 2001).

Determining, the faunistic composition and distribution patterns of phlebotomine sand flies can indicate the possible presence of autochthonous transmission and/or can aid in the incrimination of the vector species. It is essential to know the distribution of sand flies along various altitudinal gradients for continuous monitoring of VL and CL vectors in the study areas. Therefore, the present study was undertaken to determine species composition, distribution, diversity, altitudinal and ecological relationships of sand flies in representative localities between Kafta Humera (lowland) and Gondar town(highland)in northwest Ethiopia.

## 2 Material and methods

### 2.1 Study localities between Kafta Humera and Gondar

**Fig. 1:**
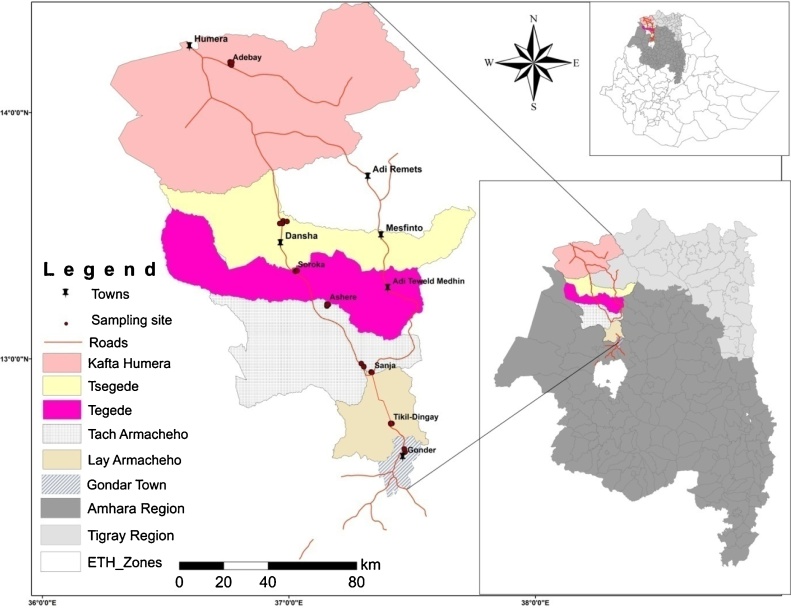
Map showing study villages on the main road from Kafta Humera to Gondar town.

The study was conducted in a transect from Kafta Humera district to Gondar town (Fig. 1) because the sites represents a wide altitudinal range in a relatively compact and well defined geographical area. For this purpose, seven localities (Adebay, Dansha, Soroka, Ashere, Sanja, Tikil-Dingay and Gondar) were selected with distinct altitudinal variations between the two major localities along a transect on the main paved road between Humera and Gondar towns (Table 1). Additional criteria for selection were accessibility, security and availability of minimal accommodation/subsistence facilities. The altitude varied from 550 m a.s.l in Kafta Humera to 2300 m a.s.l in Gondar.

**Table 1:** Physical parameters and collection site description of the localities in the study area.

Study sites	Alt (m a.s.l)	Latitudes	Longitudes	Habitat description	VL status
Adebay	650	14°11′23″N	036°45′11″E	Semi-urban, cracked mud walls, vertisols, plains, Sesame and sorghum farm, *Acacia* trees, *Balanites* trees, semi-arid	Endemic
Dansha	779	13^°^33′36″N	037^°^00′81″E	Urban, cement and mud walls houses, vertisols, plains, sesame and sorghum farm, *Acacia* trees and other mixing forest, semi-arid	Endemic
Soroka	834	13^°^21′33″N	037^°^01′56″E	Semi-urban, mud walled houses, vertisols, Sesame, sorghum and rice farms, *Acacia* trees, plains, Angereb river, semi-arid	Rarely reported
Ashere	890	13^°^13′38″N	037^°^09′30″E	Semi-urban, mud walls houses, vertisols, Sesame, sorghum and rice farms, *Acacia* trees, plains, Angereb river, semi-arid	Risk area
Sanja	1058	13^°^00′43″N	037^°^17′81″E	Urban, cement and mud walls houses, domestic animals, vertisols, Sesame, sorghum and rice farms, *Acacia* trees, plains, Angereb river, semi-arid	Risk area
Tikildingay	2027	12^°^44′23″N	037°25′10″E	Semi-urban, mud walls houses, clay soils, Eucalyptus trees, small bushes, mountainous and hilly, rocky, hyrax, highland,	Non endemic
Gondar town	2279	12^°^37′29″N	037^°^28′28″E	Urban, cement houses, clay soils, Eucalyptus trees, small bushes, mountainous, hilly, highland	Non endemic

#### 2.1.1 Adebay (Kafta Humera district)

Kafta Humera district is found in Western Tigray Zone, Northwest Ethiopia. It is situated at about 967 km from Addis Ababa. The district is mostly flat plain at altitudes of 550–699 m a.s.l. The villages are surrounded by uniform agricultural fields. Most of the natural vegetation in the Humera lowlands has been cleared for the extensive commercial agricultural practices, leaving only scattered *Acacia* and *Balanite*s trees with neem (*Azardirachta indica*) grown commonly in urban areas as shade trees. Temperature rises to a maximum average of 42 °C between April and June and falls to between 25 and 35 °C during the moderate months between June and February. Crop production is exclusively dependent on the unimodal precipitation (average annual rainfall is 400–650 mm), which runs from July to September (Gemetchu et al., 1975).

#### 2.1.2 Dansha town (Tsegede district, Western Tigray)

This is a small town situated about100kms from Setit Humera and 150 km from Gondar at altitude range of 700–799 m a.s.l. It is semi-arid with vertisol type of soil. Sesame and livestock are main the income sources in the area. The vegetation comprises *Acacia* trees and mixed forest.

#### 2.1.3 Soroka (Tegede district, North Gondar Zone, Amhara region)

It is a semi- urban area about 125 km from Setit Humera and 125 km from Gondar town. It is located at altitude 800–849 m a.s.l. Livestock and sesame and sorghum farming are the main economic activities. *Acacia*, *Balanites* and *Boswellia* (incense) trees are common, although much of the natural vegetation has been cleared for farming, fuelwood and constructions (huts and fences). Angerib River passes through the village to join the Atbara River system in the eastern Sudan.

#### 2.1.4 Ashere (Tach Armachiho district, North Gondar Zone, Amhara region)

This is a small town about 155 km from Setit Humera and 95 km from Gondar. It is located at altitudes of 850–920 m a.s.l. The vegetation and economic activities are similar to other small towns described above. Ashere is semi-arid with warm temperature during the dry season and it has vertisol soil type.

#### 2.1.5 Sanja town (Tach Armachiho district, North Gondar Zone, Amhara region)

It is a small town situated at 950–1100 m a.s.l and 200 km from Setit Humera and 50 km from Gondar town. The annual rainfall is 300 mm to 750 mm with slight rains in April and May and heavy rains in July and August. Sanja town is semi-arid area with temperature ranging from 29 °C to 31 °C. Livestock and cash crop production are the main economic activities.

#### 2.1.6 Tikil-Dingay(Lay Armachiho district, North Gondar Zone, Amhara region)

It is a small semi-urban village at about 1950–21000 m a.s.l and located about 225 km from Setit Humera and 25 km from Gondar. It is surrounded by a mountainous ridge where rock hyraxes are occasionally observed. The natural vegetation is almost non-existent, with *Eucalyptus* trees and shrubs being common on the periphery.

#### 2.1.7 Gondar Town (North Gondar, Amhara region)

It is situated about 250 km from Setit Humera and with altitudes of 2200–2300 m a.s.l. The town is mountainous with many *Eucalyptus* trees.

### 2.2 Sand fly collections

**Fig. 2:**
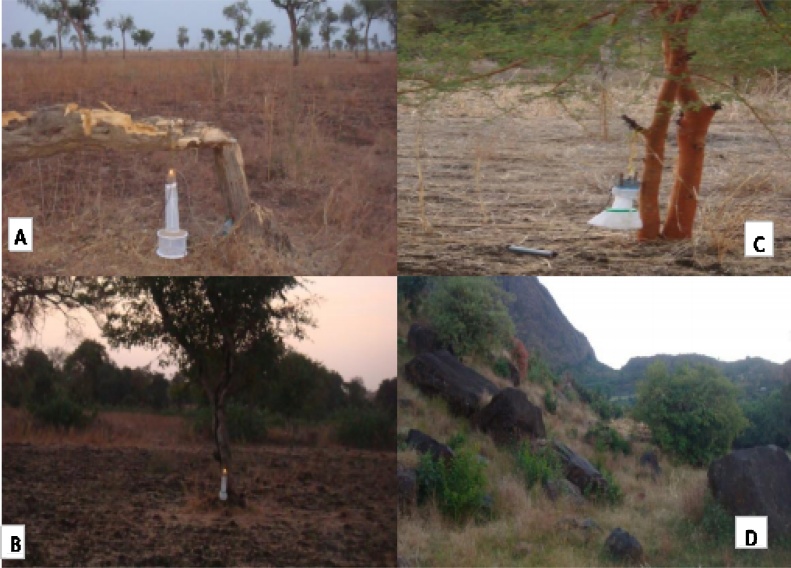
Sand flies collection sites from Humera to Gondar transect. A) Adebay(Sesame farm), B) Soroka(Rice field), C) Ashere(Acacia trees), D) Tikil-Dingay(Hyrax habitat).

In each of the seven study sites, sand flies were collected using standard CDC light traps (LTs) and sticky traps (STs) during the active sand fly season from December 2012 to May 2013 (6 months). Sand fly collections were performed for 1–2 nights per month in each study site during the first and second weeks. A total of 362 LTs were used for 72 nights during the six months. Traps were suspended 0.4–0.5 m above the ground level and were deployed about 100–200 m apart. Traps were placed in different habitats in each locality based on the type of geographical setting. In Adebay, two LTs were placed in each habitat: inside the village, periphery of the village and agricultural farm fields. In Dansha, Soroka, Ashere and Sanja localities, two LTs were deployed in the domestic and peri-domestic habitat (near houses and animal shelter), in farm fields (close to the small towns) and in the forest (*Acacia* trees). In the same way sand flies in Tikil Dingay were sampled from peridomestic, forest and rocky hyrax habitats while in Gondar town collection sites were around peridomestic and rocky gorges (Fig. 2).

A4-sized white polypropylene boards were coated with sesame oil for use as sticky traps (STs) and sand flies were collected from a total of 2, 589 STs in 72 nights during the six months observation period. For indoor collections, five STs per house in 2 houses per locality per night were used. These were tied serially about 30–40 cm apart with a nylon string and suspended by the wall. For outdoor collections, 10 STs were placed per site per night, which were also tied serially as above with a nylon string and were placed vertically over different habitats in each locality. STs were set in various locations at each study site. In Adebay ST collections were made from indoors, around human dwellings (inside and periphery of the village) and agricultural farm. In Dansha, Soroka, Ashere and Sanja localities, STs were placed indoors, domestic and peridomestic habitats, agricultural farms, termite hills and *Acacia* trees. In Tikil Dingay, STs were set indoors, inside village, peridomestic and rocky habitats. In Gondar town, STs were deployed in peridomestic habitats and gorges. Sand flies were collected from the sticky traps with needles or *Acacia* thorns.

Both LTs and STs were placed at 18:00 h and were collected in the following morning. Sand flies were brought to the field laboratory where they were transferred from cages, to test tubes and stored in absolute alcohol until later mounting and identification to species.

### 2.3 Processing, mounting and identification of sand flies

All collections of sand flies were preserved in absolute alcohol. The head and abdominal tip of each sand fly were removed and mounted on slides using a drop of Hoyer medium for species identification. All phlebotomine sand flies (males and females) from LTs and STs were slide mounted and identified to species based on cibarial and pharyngeal armature as well as spermathecae of females and external genitalia of males using morphological keys (Quate, 1964, Abonnenc and Minter, 1965, Lewis, 1982, Lane and Fritz, 1986, Lane, 1993, Gebre-Michael and Medhin, 1997).

### 2.4 Data analysis

All data were entered in excel sheet and statistical analysis was performed with SPSS version 20.0 software (SPSS Inc., Chicago, IL, USA). Nonparametric Kruskal-Wallis test (KW) was used to compare sand fly abundance between traps. Spearman’s nonparametric correlation was performed between densities of *Phlebotomus* species and altitude. Shannon-Weiner species diversity index and Jaccard’s coefficient was used to estimate species diversity and similarity between altitudes, respectively (Doha and Samy, 2010, Belen and Alten, 2011). Species diversity is a measure of diversity within an ecological community that encompasses both species richness and evenness of species abundance (Hamilton, 2005). Species richness is the number of species in a particular area or environment, whereas, species evenness refers how close each species in number (abundance) are in the same area (Hamilton, 2005, Simsek et al., 2007).

## 3 Results

### 3.1 Sand fly species composition

A total of 89,044 sand flies (41,798 males and 47, 246 females) were collected using CDC light traps and sticky traps from the seven localities/towns (Table 2). Twenty two species belonged to 11 species in the genus *Phlebotomus* and the same number of species in *Sergentomyia were recorded .* Among these, *S. clydei* (25.87%), *S. schwetzi* (25.21%), *S. africana* (24.65%), *S. bedfordi* (8.89%), *P. orientalis* (6.43%), and *S.antennata* (4.8%) were the most prevalent species. The remaining 10 *Phlebotomus* species and six *Sergentomyia* were either less frequent or very rare.

**Table 2:** Number and relative abundance of sand flies species in the study areas based on LTs and STs (Dec. 2012-May 2103).

Species	LTs		STs		Overall Total (%)
	M	F	M	F	
*P. orientalis*	2374	1066	1527	759	5726(6.43)
*P. longipes*	36	28	3	0	67(0.08)
*P. gibiensis*	6	3	0	0	9(0.01)
*P. papatasi*	226	116	197	101	640(0.72)
*P. duboscqi*	47	32	54	39	172(0.19)
*P. bergeroti*	67	39	47	34	187(0.21)
*P. saevus*	1	0	0	0	1(0.001)
*P. alexanderi*	8	8	4	0	20(0.02)
*P. rodhaini*	28	33	10	5	76(0.09)
*P. lesleyae*	178	100	178	100	556(0.62)
*P. heischi*	47	38	8	7	100(0.11)
*S. africana*	5694	6554	4462	5237	21,947(24.65)
*S. bedfordi*	1089	3073	1048	2702	7912(8.89)
*S. schwetzi*	6126	5909	5397	5013	22,445(25.21)
*S. squamipleuris*	41	803	47	515	1406(1.58)
*S. clydei*	6985	6604	4923	4520	23,032(25.87)
*S. antennata*	548	1981	359	1398	4286(4.81)
*S. dubia*	0	134	0	151	285(0.32)
*S. adleri*	22	87	11	49	169(0.19
*S. collarti*	0	4	0	0	4(0.004)
*S. calcarata*	0	3	0	0	3(0.003)
*S. adami*	0	0	0	1	1(0.001)
**Total**	**23,523**	**26,615**	**18,275**	**20,631**	**89,044(100)**

The Kruskal-Wallis test indicated that the abundance of males and females phlebotomine sand flies were significantly different between LTs and STs (P < 0.001). LT collected more number of sand flies than STs from Kafta Humera to Gondar (Table 2). The distribution and abundance of males and females phlebotomine sand flies were showed variation between study sites and altitudes (Table 3). *Sergentomyia africana*, *S. bedfordi*, *S. schwetzi*, *S. squamipleuris* and *S. clydei* were recorded from all study areas and altitudes in the transect. On the other hand, *P. orientalis*, *P. rodhaini* and *P. bergeroti*, were the next wide speared species but absent in two of the study sites at higher altitudes in Tikil-Dingay and Gondar town (>2000 m a.s.l). The numbers of species were much higher in the low altitude areas than the high altitude localities. Thus, 17 species were recorded in Adebay (Humera) (550–699 m a.s.l) and 12–14 species in Dansha, Soroka, Ashere and Sanja (700–1100 m a.s.l). At higher altitudes, less number of species were recorded where only three *Phlebotomus* species namely *P. longipes*, *P. gibiensis* and *P. saevus* were documented, the only exception was *P. duboscqi* with a single specimen collected in Tikil-Dingay (Table 3).

**Table 3:** Number (N) and relative abundance (%) of sand fly species collected in different localities between the lowland (Adebay-Humera) and highland (Gondar) districts based on LTs and STs (Dec. 2012- May 2013).

Spp	Adebay		Dansha		Soroka		Ashere		Sanja		Tikildingay		Gondar			
	550–699 m a.s.l		700–799 m a.s.l		800–849 m a.s.l		850–949 m a.s.l		950–1100 m a.s.l		1950–2099 m a.s.l		2200–2300 m a.s.l		Total	
	N (%)		N (%)		N (%)		N (%)		N (%)		N (%)		N (%)		N (%)	
	M	F	M	F	M	F	M	F	M	F	M	F	M	F	M	F
*P. orientalis*	3496(3.93)	1657(1.86)	151(0.17)	43(0.05)	138(0.15)	62(0.07)	96(0.11)	58(0.07)	20(0.02)	5(0.01)	0(0.0)	0(0.0)	0(0.0)	0(0.0)	3901(4.38)	1825(2.05)
*P. longipes*	0(0.0)	0(0.0)	0(0.0)	0(0.0)	0(0.0)	0(0.0)	0(0.0)	0(0.0)	0(0.0)	0(0.0)	15(0.02)	9(0.01)	24(0.03)	19(0.02)	39(0.04)	28(0.03)
*P. gibiensis*	0(0.0)	0(0.0)	0(0.0)	0(0.0)	0(0.0)	0(0.0)	0(0.0)	0(0.0)	0(0.0)	0(0.0)	3(0.003)	3(0.003)	3(0.003)	0(0.0)	6(0.01)	3(0.003)
*P. papatasi*	407(0.46)	211(0.24)	11(0.01)	4(0.004)	3(0.003)	1(0.001)	2(0.002)	1(0.001)	0(0.0)	0(0.0)	0(0.0)	0(0.0)	0(0.0)	0(0.0)	423(0.48)	217(0.24)
*P. duboscqi*	95(0.11)	69(0.08)	5(0.01)	2(0.002)	0(0.0)	0(0.0)	0(0.0)	0(0.0)	0(0.0)	0(0.0)	1(0.001)	0(0.0)	0(0.0)	0(0.0)	101(0.11)	71(0.08)
*P. bergeroti*	94(0.11)	64(0.07)	11(0.01)	4(0.004)	4(0.004)	2(0.002)	4(0.004)	2(0.002)	0(0.0)	1(0.001)	0(0.0)	0(0.0)	0(0.0)	0(0.0)	113(0.13)	73(0.08)
*P. saevus*	0(0.0)	0(0.0)	0(0.0)	0(0.0)	0(0.0)	0(0.0)	0(0.0)	0(0.0)	0(0.0)	0(0.0)	1(0.001)	0(0.0)	0(0.0)	0(0.0)	1(0.001)	0(0.0)
*P. alexanderi*	12(0.01)	6(0.01)	1(0.001)	0(0.0)	0(0.0)	2(0.002)	0(0.0)	0(0.0)	0(0.0)	0(0.0)	0(0.0)	0(0.0)	0(0.0)	0(0.0)	13(0.01)	8(0.01)
*P. rodhaini*	10(0.0)	9(0.01)	4(0.004)	4(0.00)	8(0.01)	9(0.01)	14(0.02)	12(0.010	2(0.002)	4(0.004)	0(0.0)	0(0.0)	0(0.0)	0(0.0)	38(0.04)	38(0.04)
*P. lesleyae*	356(0.4)	200(0.22)	0(0.0)	0(0.0)	0(0.0)	0(0.0)	0(0.0)	0(0.0)	0(0.0)	0(0.0)	0(0.0)	0(0.0)	0(0.0)	0(0.0)	356(0.4)	200(0.22)
*P. heischi*	8(0.01)	4(0.004)	0(0.0)	0(0.0)	35(0.04)	30(0.03)	5(0.005)	6(0.01)	7(0.01)	5(0.01)	0(0.0)	0(0.0)	0(0.0)	0(0.0)	55(0.06)	45(0.05)
*S. africana*	2068(2.32)	1938(2.18)	2655(2.98)	3097(3.48)	2478(2.78)	3114(3.5)	1505(1.69)	1516(1.7)	1270(1.42)	1800(2.02)	148(0.17)	276(0.31)	32(0.04)	50(0.06)	10,156(11.41)	11,791(13.24)
*S. bedfordi*	197(0.22)	302(0.34)	818(0.92)	1803(2.03)	426(0.48)	1221(1.37)	257(0.29)	862(0.97)	367(0.410	1365(1.53)	58(0.07)	189(0.21)	14(0.02)	33(0.04)	2137(2.40)	5775(6.49)
*S. schwetzi*	3177(3.57)	2882(3.24)	3182(3.57)	3174(3.57)	3065(3.44)	2636(2.96)	1264(1.42)	1202(1.34)	766(0.86)	927(1.04)	60(0.07)	88(0.1)	9(0.01)	13(0.01)	11,523(12.94)	10,922(12.27)
*S. squamipleuris*	27(0.03)	300(0.34)	12(0.01)	231(0.26)	5(0.01)	113(0.13)	2(0.002)	128(0.14)	38(0.04)	482(0.54)	2(0.002)	49(0.06)	2(0.002)	15(0.02)	88(0.1)	1318(1.48)
*S. clydei*	3630(4.08)	3110(3.49)	3308(3.72)	3107(3.49)	2657(2.98)	2742(3.08)	1245(1.4)	1225(1.38)	1027(1.15)	894(1.00)	36(0.04)	41(0.05)	5(0.01)	5(0.01)	11,908(13.37)	11,124(12.49)
*S. antennata*	100(0.11)	301(0.34)	420(0.47)	1096(1.23)	120(0.13)	600(0.67)	66(0.07)	527(0.59)	197(0.22)	821(0.92)	4(0.004)	34(0.04)	0(0.0)	0(0.0)	907(1.02)	3379(3.79)
*S. dubius*	0(0.0)	82(0.09)	0(0.0)	172(0.19)	0(0.0)	4(0.004)	0(0.0)	13(0.01)	0(0.0)	14(0.02)	0(0.0)	0(0.0)	0(0.0)	0(0.0)	0(0.0)	285(0.32)
*S. adleri*	20(0.02)	71(0.08)	10(0.01)	59(0.07)	0(0.0)	2(0.002)	1(0.001)	2(0.002)	2(0.002)	2(0.002)	0(0.0)	0(0.0)	0(0.0)	0(0.0)	33(0.04)	136(0.15)
*S. colarati*	0(0.0)	4(0.004)	0(0.0)	0(0.0)	0(0.0)	0(0.0)	0(0.0)	0(0.0)	0(0.0)	0(0.0)	0(0.0)	0(0.0)	0(0.0)	0(0.0)	0(0.0)	4(0.004)
*S .calcaratus*	0(0.0)	0(0.0)	0(0.0)	0(0.0)	0(0.0)	0(0.0)	0(0.0)	3(0.003)	0(0.0)	0(0.0)	0(0.0)	0(0.0)	0(0.0)	0(0.0)	0(0.0)	3(0.003)
*S. adami*	0(0.0)	0(0.0)	0(0.0)	0(0.0)	0(0.0)	0(0.0)	0(0.0)	0(0.0)	0(0.0)	0(0.0)	0(0.0)	1(0.001)	0(0.0)	0(0.0)	0(0.0)	1(0.001)
Total	13,697(55)	11,210(45)	10,588(45.3)	12,796(54.7)	8,939(45.9)	10,538(54.1)	4461(44.5)	5557(55.5)	3696(36.9)	6320(63.1)	328(32.2)	690(67.8)	89(39.7)	135(60.3)	41,798(46.9)	47,246(53.1)

### 3.2 Species diversity

There were differences in species diversity of phlebotomine sand flies as indicated by the values of Shannon-Wiener index (H), evenness (E), and richness (S) of the sand fly fauna along the study areas (Table 4). Higher species diversity and species richness was observed in Adebay but it showed lower species evenness as compared to other localities with the exception of Sorkoa in the case of ST collections of both sexes. For LTs collections, lower species evenness was found in Soroka for both male and female sand flies. The highest species evenness was observed in Gondar for both sexes and collection methods. In Gondar, there was low species diversity and richness of both male and female sand flies from collections of STs and only in females from LT catches.

In terms of altitude, there was also a significant difference in the diversity of the sand fly fauna as indicated by the values of Shannon-Weiner index (H) (Table 4). The richness and diversity was maximal between 550 and 699 m a.s.l altitude in both collection methods (Male: LT: H = 1.72, E = 0.66, S = 17 and ST: H = 1.73, E = 0.64, S = 15; females: LT: H = 1.87, E = 0.64, S = 15 and ST: H = 1.87, E = 0.64, S = 14). In both traps, the least species diversity was observed at 700–849 m a.s.l for males (LT: H = 1.47, ST: 1.42) and 1950–2300 m a.s.l for females (LT: H = 1.61, ST: H = 1.53). Relatively low species richness and high evenness was also observed at altitude 1950–2300 m a.s.l for both sexes and LTs and STs.

**Table 4:** The Shannon-Weiner diversity index (H), evenness (E) and richness(S) for the sand fly in different districts and at different altitude ranges in the northwest Ethiopia.

Localities	LTs						STs					
	H′		S		E		H′		S		E	
	M	F	M	F	M	F	M	F	M	F	M	F
Adebay	1.72	1.87	15	17	0.64	0.66	1.73	1.87	15	14	0.64	0.71
Dansha	1.5	1.69	12	13	0.60	0.66	1.48	1.71	9	9	0.67	0.78
Soroka	1.42	1.59	11	13	0.59	0.62	1.33	1.56	8	9	0.64	0.71
Ashere	1.47	1.77	12	14	0.59	0.67	1.37	1.61	7	9	0.70	0.73
Sanja	1.49	1.75	10	12	0.65	0.70	1.56	1.72	9	8	0.71	0.83
Tikil-Dingay	1.56	1.59	10	8	0.68	0.76	1.45	1.53	7	7	0.75	0.79
Gondar	1.53	1.55	7	6	0.79	0.87	1.26	1.4	5	5	0.78	0.87

Altitudes (m a.s.l)	H′		S		E		H′		S		E	
	M	F	M	F	M	F	M	F	M	F	M	F
550–699	1.72	1.87	15	17	0.64	0.66	1.73	1.87	15	14	0.64	0.71
700–849	1.47	1.66	13	15	0.57	0.61	1.42	1.65	11	10	0.59	0.72
850–1100	1.5	1.78	11	14	0.63	0.67	1.47	1.68	9	8	0.67	0.81
1950–2300	1.61	1.61	10	8	0.7	0.77	1.44	1.53	7	7	0.74	0.79

Key: M = Male, F = Female, H' = Shannon-Weiner index, S = Species richness, E = Evenness.

**Table 5:** The similarity of phlebotomine sand flies of Jaccards coefficient between altitudes in the northwest Ethiopia.

Altitudes (m a.s.l)	Jaccards coefficient							
	M				F			
	550–699	700–849	850–1100	1950–2300	550–699	700–849	850–1100	1950–2300
550–699	1	0.87	0.8	0.39	1	0.88	0.72	0.32
700–849	0.87	1	0.92	0.44	0.88	1	0.81	0.35
850−1100	0.8	0.92	1	0.4	0.72	0.81	1	0.38
1950–2300	0.39	0.44	0.4	1	0.32	0.35	0.38	1

The sand fly species similarity result of Jacard’s coefficient (IJacard) between the altitudes is presented in Table 5. The similarity in sand fly species was 87% for males and 88% for females between the altitude 550–699 m a.s.l and 700–849 m a.s.l. The similarity between 700 and 849 m a.s.l and 850–1100 m a.s.l reached 92% for males and 81% for females and between 700 and 849 m a.s.l and 1950–2300 m a.s.l was 44% and 35% for males and females respectively. The similarity between 850 and 1100 m a.s.l and 1950–2300 m a.s.l reached 40% and 38% for males and females respectively (Table 5).

*P. orientalis* distributed between the altitudes of 550 m a.s.l and 1100 m a.s.l and its distribution showed a high negative correlation with altitude (LT: Male: r = −0.71, female: r = −0.67; ST: male: r = −0.47′ female: r = −0.49). Similarly, distribution of both sexes of the three sympatric and related species *P .papatasi*, *P. bergeroti* and *P. duboscqi* showed significant negative correlation with altitude for both traps. On the other hand, presence of *P. longipes* was significantly and positively correlated with altitudes in LT collections (Male: r = 0.45, female: r = 0.40) (Table 6).

**Table 6:** The Spearman’s correlation coefficient between densities of sand flies and altitude.

spp	LTs				STs			
	M		F		M		F	
	r	P	r	P	r	P	r	P
*P. orientalis*	−0.70	<0.001	−0.67	<0.001	−0.47	<0.001	−0.49	<0.001
*P. longipes*	0.45	<0.001	0.40	<0.001	0.13	0.048	–	–
*P. gibiensis*	0.21	0.004	0.12	0.123	–	–	–	–
*P. papatasi*	−0.61	<0.001	−0.50	<0.001	−0.58	<0.001	−0.51	<0.001
*P. duboscqi*	−0.47	<0.001	−0.41	<0.001	−0.43	<0.001	−0.41	<0.001
*P. bergeroti*	−0.45	<0.001	−0.40	<0.001	−0.44	<0.001	−0.35	<0.001
*P. saevus*	0.08	0.281	–	–	–	–	–	–
*P. alexanderi*	−0.26	<0.001	−0.25	0.001	−0.20	0.002	–	–
*P. rodhaini*	−0.19	0.012	−0.16	0.032	−0.04	0.501	−0.10	.103
*P. lesleyae*	−0.49	<0.001	−0.49	<0.001	−0.43	<0.001	−0.42	<0.001
*P. heischi*	−0.14	0.067	−0.11	0.124	−0.14	0.028	−0.03	.663
*S. africana*	−0.61	<0.001	−0.55	<0.001	−0.52	<0.001	−0.41	<0.001
*S. bedfordi*	−0.32	<0.001	−0.19	0.011	−0.28	<0.001	−0.20	0.002
*S. schwetzi*	−0.74	<0.001	−0.67	<0.001	−0.61	<0.001	−0.61	<0.001
*S. squamipleuris*	−0.11	0.127	−0.12	0.122	−0.05	0.408	−0.08	0.199
*S. clydei*	−0.74	<0.001	−0.73	<0.001	−0.57	<0.001	−0.57	<0.001
*S. antenata*	−0.33	<0.001	−0.28	<0.001	−0.21	0.001	−0.25	<0.001
*S. dubia*	–	–	−0.32	<0.001	–	–	−0.40	<0.001
*S. adleri*	−0.26	<0.001	−0.26	<0.001	−0.17	0.008	−0.26	<0.001
*S. collarti*	–	–	−0.17	0.025	–	–	–	–
*S .calcarata*	–	–	0.024	0.749	–	–	–	–
*S. adami*	–	–	–	–	–	–	0.07	0.271
Total	−0.77^**^	<0.001	−0.67^**^	<0.001	−0.64^**^	<0.001	−0.583^**^	<0.001

## 4 Discussion

Understanding the distribution of sand fly vectors is very essential to determine transmission dynamics of leishmaniasis. In this regard, this study documented the species composition, diversity and altitudinal distribution of phlebotomine of sand flies in different districts along an altitudinal transect from low (550 m a.s.l) to high altitudes (2300 m a.s.l) between Humera and Gondar Town in northwest Ethiopia which is the first of its kind in the country. Although 22 species of sand flies were found, some of the species were already known to occur in the lowlands of Humera and Metema districts (Gemetchu et al., 1975, Gemetchu, 1983, Gebre-Michael et al., 2010, Yared et al., 2017).

*Phlebotomus orientalis,* a vector of VL in the study areas, showed strong negative correlation with altitudes. This species occurred in localities between the altitude ranges of 550 m a.s.l and 1100 m a.s.l, and was absent in two high altitude (1950–2300 m a.s.l) localities (Tikil-Dingay and Gondar) with mountainous and hilly topography. It was the most abundant in Adebay, a locality with the lowest altitude. This species was previously reported from northwest and northeast Ethiopia (Ashford, 1974, Gebre-Michael et al., 2004a, Gebre-Michael et al., 2007, Gebre-Michael et al., 2010, Yared et al., 2017). The distribution of this species has wide climatic and altitudinal variability in East Africa (Gebre-Michael et al., 2004b) occurring at altitudinal ranges of 200–2200 m a.s.l, with annual temperature of 16–36 °C and annual rain fall of 180–1050 mm. A study based on logistic regression model indicated that mean annual maximum daily temperature and soil type are the most important ecological determinants for the distribution of *P. orientalis* in Sudan (Thomson et al., 1999) although a single soil type (vertisol) did not come out as important in Ethiopia, as many soil types were associated with the distribution of *P. orientalis* (Gebre-Michael et al., 2004b). Based on the risk model analysis, Tsegaw et al. (2013) showed that the lowlands of north western Ethiopia fall in the high and very high risk areas of VL.

*Phlebotomus longipes* and *P. gibiensis* had a significant positive correlation with altitude. The occurrence of *P. longipes* in two localities (Tikil-Dingay and Gondar) at high altitudes was not unexpected and had previously been recorded in Gondar Town. The species is one of the vectors of CL (*L. aethiopica*) in much of the Ethiopian highlands (Foster, 1972, Ashford et al., 1973, Hailu et al., 2006). However, the occurrence of *P. saevus* and *P. gibiensis* in localities with over 2000 m a.s.l was unexpected. The two species have so far been recorded at altitudes ranging between 700 and 1700 m a.s.l in the Rift valley and outside the Rift valley associated with rocky habitats of hyraxes (Ashford, 1974, Balkew et al., 2002), *P.saevus* was found infected with *L. tropica* (CL) in the Awash Valley (Gebre-Michael et al., 2004a).

The occurrence of the three closely related species, *P. papatasi*, *P. duboscqi* and *P. bergeroti* sympatrically in localities of the lower altitude (550–1100 m a.s.l) was in agreement with previous reports from the region (Gebre-Michael et al., 2010, Yared et al., 2017) and the neighbouring Sudan (Hoogstraal and Heyneman, 1969). However, the occurrence of *P. duboscqi* in Tikil-Dingay of high altitude, though only a single specimen, was unusual. The highest altitude so far recorded for *P. duboscqi* in Ethiopia was about 1800 m a.s.l in Arbaya (northern Ethiopia) (Ashford, 1974). *P. papatasi* and *P.dubocqui* species are known vectors CL due to *L. major* in sub-Saharan Africa, and *P. papatasi* is more important in North Africa, the Mediterranean and Asia (WHO, 2010). *L. major* is so far unknown in northwest Ethiopia, while it is an important problem in Sudan, West Africa, North Africa and the Middle East (WHO, 2010). In the present study, *P. papatasi* was the second abundant among the three species. This species was found at altitudinal ranges of 550–1100 m a.s.l and occurred with decreasing altitudinal gradient along the study areas. Similarly, in Morocco *P. papatasi* showed a negative correlation with altitude (r = −0.82) (Guernaoui et al., 2006) being present at altitudes between 400 and 600 m a.s.l, and absent from 1200 m a.s.l (Guernaoui et al., 2006). In Saudi Arabia, high densities of *P. papatasi* were found between 800 and 1200 m a.s.l (Doha and Samy, 2010).

The record of *P. rodhaini* in all localities (<1100 m a.s.l) except the highlands is significant although not very numerous. This species has been recently found infected with *L. donovani* in eastern Sudan close to the Ethiopian border (Elnaiem et al., 2011). The two closely related species, *P. lesleyae* and *P. heischi* were sympatric in the lowest altitude village, Adebay (Humera), where the former was more abundant, but *P. heischi* was allopatric occurring up to Sanja (1100 m a.s.l). The two species have previously been recorded in northwest Ethiopia (Gemetchu et al., 1975, Yared et al., 2017) and at least one of them *(P. heischi*) as high as 1800 m a.s.l in Arbaya (northern Ethiopia) (Ashford, 1974) and is also known to bite humans (Hoogstraal and Heyneman, 1969), but their role as vectors of leishmaniais is not yet known.

The species of *Sergentomyia* accounted for 91.5% of the entire sand fly collection (n = 89,044), with only 8.5% from the genus *Phlebotomus* using both LT and ST traps. This showed that the population densities of the *Phlebotomus* species were relatively very low in numbers as compared with the species of the genus *Sergentomyia*. *Sergentomyia* spp. are generally rich in species and number in Africa south of Sahara and Southeast Asia and often overwhelm the *Phlebotomus* species (Lane, 1993). The reverse is true in most of the Palaearctic region (Simsek et al., 2007). *Sergentomyia* species are not associated with human disease transmission, although some species are known to bite humans (Lane, 1993). Previous work, by Gebre-Michael and Lane (1996) found 17 *Sergentomyia* species between the altitude ranges of 1400–1550 m a.s.l in southern Ethiopia. Some of the species recorded in the present study have also been previously reported from Sudan (Quate, 1964, Hoogstraal and Heyneman, 1969). In, contrary to the distribution of *Phlebotomus* species, the five most common *Sergentomyia* species (*S. schwetzi*, *S. clydei*, *S. africana*, *S. bedfordi*, and *S. squamipleuris*) were found in all localities/towns from the low to high altitude, although at lower densities with increased altitude. This indicates altitude alone may not be a selective factor. Ashford (1974) reported the greatest number of *Sergentomyia* species at altitudes below 1300 m a.s.l.

A clear difference was observed in the species richness and diversity of sand fly fauna, in both the localities and altitudes. The species richness and diversity was the highest in Kafta Humera districts (Adebay village) with the lowest altitude (550–699 m a.s.l) where 17 of 22 species recorded were present here. Sand fly distributions could be affected by climate change and by the variation of ecological conditions due to the changing land-use and settlements (Guernaoui et al., 2005). Previous studies in southern Turkey, the High-Atlas Mountains (Morocco) and Saudi Arabia have revealed that species composition and diversity are directly related to altitudinal variations (Guernaoui et al., 2006, Simsek et al., 2007, Doha and Samy, 2010). For instance, in southern Turkey, the highest species diversity was found at altitudinal ranges of 400–600 m a.s.l whereas high species richness was recorded in the range of 0–200 m a.s.l (Simsek et al., 2007). In the High-Atlas Mountains, high species diversity and richness has been found between at altitudinal ranges of 800–999 m a.s.l (Guernaoui et al., 2006), whereas Doha and Samy (2010) reported high species richness and diversity at altitudinal range of 800–1200 m a.s.l in Saudi Arabia. In contrast, Faraj et al. (2013) have recorded a high diversity at 1500 m a.s.l in Saharan area in Morocco. There was no significant correlation between the abundance of sand flies and altitude in Cukurova region of Turkey (Belen and Alten, 2011). The Shannon-Weinner index indicated no difference between the diversity and abundance of sand flies at different altitudes. This diversity and evenness reached maximum values at 500 m a.s.l (Belen and Alten, 2011). Others suggested that altitude has an influence upon the spatial distribution and density of the sand fly vectors (Guernaoui et al., 2006).

Sand fly distributions are highly different within different altitude ranges in the study area. Their distributions are highly dependent on local environmental factors such as temperature, rain fall and relative humidity as well as physical factors like geographical barriers and habitat availability and also abiotic factors such as the distribution and abundance of vertebrate hosts (Cross et al., 1996, Ghosh et al., 1999). Our study also revealed that altitude influences the spatial distribution of sand flies in the study area. However, it seems that altitude by itself is not considered as ecological factor but the associated climatic variability and other biotic and abiotic factors of the environment are highly correlated with altitudinal gradients (Simsek et al., 2007). Furthermore, the geographical and ecological diversity of the region provides large number of sand flies. Hence, the result of this study has clear implications for our general understanding of the biology of sand fly species and their distributions as well as for predicting the risk of VL and CL.

In conclusion, the sand fly fauna in northwest Ethiopia with various altitude range are diverse with 22 species. Our data showed that *P. orientalis* occurred between altitude 500–1100 m a.s.l in northwest Ethiopia. As a result, the area could be at high risk of VL possibility of new outbreaks in the area. However, no *P. orientials* was recorded between altitude 1950–2300 m a.s.l due to mountainous and hilly topography. The presence of species of subgenus *Phlebotomus* (*P. papatasi, P. duboscqi* and *P. bergeroti*) in the area revealed that the epidemiological importance of these species in the region and underscores the need of further studies, such as investigations into vector competence. *Phlebotomus longipes* is considered to be proven vector of *L. aethiopica* in the country and this species was recorded in the highland area in Tikil-Dingay and Gondar town. Reservoir hosts are also present and the protozoon parasites could eventually be integrated into this complex environment and there is high possibility to produce the diseases in humans in the area. This observation may contribute to the understanding of leishmaniasis transmission in northwest Ethiopia.

## Competing interest

The authors declare that they have no competing interests.

## References

[bib0005] Abonnenc E., Minter D.M. (1965). Bilingual key for the identification of Sand flies of the Ethiopian region. Cah. ORSTOM Entomol. Med..

[bib0010] Alvar J., Bashaye S., Argaw D., Cruz I., Aparicio P., Kassa A., Orfanos G., Parreño F., Babaniyi O., Gudeta N., Cañavate C., Bern C. (2007). Kala-Azar outbreak in Libo Kemkem, Ethiopia: epidemiologic and parasitologic assessment. Am. J. Trop. Med. Hyg..

[bib0015] Alvar J., Vélez I.D., Bern C., Herrero M., Desjeux P., Cano J., Jannin J., den Boer M. (2012). Leishmaniasis worldwide and global estimates of its incidence. PLoS One.

[bib0020] Ashford R.W., Bray M.A., Hutchinson M.P., Bray R.S. (1973). The epidemiology of cutaneous leishmaniasis in Ethiopia. Trans. R. Soc. Trop. Med. Hyg..

[bib0025] Ashford T.W. (1974). Sand flies (Diptera, Phlebotomidae) from Ethiopia: taxonomic and biological notes. J. Med. Entomol..

[bib0030] Balkew M., Gebre-Michael T., Berhe N., Ali A., Hailu A. (2002). Leishmaniasis in the middle course of the Ethiopian Rift valley: II. Entomological observations. Ethiop. Med. J..

[bib0035] Belen A., Alten B. (2011). Seasonal dynamics and altitudinal distributions of sand fly (Diptera: Psychodidae) populations in a cutaneous leishmaniasis endemic area of the Cukurova region of Turkey. J. Vector Ecol..

[bib0040] Cross E.R., Newcomb W.W., Tucker C.J. (1996). Use of weather and remote sensing to predict the geographic and seasonal distribution of *Phlebotomus papatasi* in south-west Asia. Am. J. Trop. Med. Hyg..

[bib0045] Deribe K., Meribo K., Gebre T., Hailu A., Ali A., Aseffa A., Davey G. (2012). The burden of neglected tropical diseases in Ethiopia, and opportunities for integrated control and elimination. Parasites Vectors.

[bib0050] Desjex P. (2001). Worldwide increasing risk factors for leshmaniasis. Med. Microbiol. Immunol..

[bib0055] Doha S.A., Samy A.M. (2010). Bionomics of phlebotomine sand flies (Diptera Psychodidae) in the province of Al-Baha, Saudi Arabia. Mem. Inst. Oswaldo Cruz..

[bib0060] Elnaiem D.A., Hassan K.H., Ward R.D. (1999). Association of *Phlebotomus orientalis* and other sand flies with vegetation types in the Eastern Sudan focus of kala-azar. Med. Vet. Entomol..

[bib0065] Elnaiem D.E., Hassan H.K., Osman O.F., Maingon R.D., Killick-Kendrick R., Ward R.D. (2011). A possible role for *Phlebotomus* (*Anaphlebotomus*) *rodhaini* (Parrot, 1930) in transmission of *Leishmania donovani*. Parasite Vectors.

[bib0070] Faraj C., Adlaoui E., Ouahabi S., El Kohli M., El Rhazi M., Lakraa L., Ameur B. (2013). Distribution and bionomic of sand flies in five ecologically different cutaneous leishmaniasis foci in Morocco. SRN Epidemiol..

[bib0075] Foster W.A. (1972). Studies on leishmaniasis in Ethiopia. III: resting and breeding sites, flight behavior, and seasonal abundance of *Phlebotomus longipes* (Diptera: psychodidae). Ann. Trop. Med. Parasitol..

[bib0080] Gebre-Michael T., Lane R.P. (1996). The roles of *Phlebotomus martini* and *P. celiae* (Diptera Phlebotominae) as vectors of visceral leishmaniasis in the Aba Roba focus, Southern Ethiopia. Med. Vet. Entomol..

[bib0085] Gebre-Michael T., Medhin G. (1997). Morphometric separation of female of Phlebotomus (Phlebotomus) dudoscqi and P. (P.). bergeroti (Diptera: psychodidae). J. Med. Entomol..

[bib0090] Gebre-Michael T., Balkew M., Ali A., Ludovisi A., Gramiccia M. (2004). The isolation of *Leishmania tropica* and *L. aethiopica* from *Phlebotomus* (*Paraphlebotoms*) species (Diptera: Psychodidae) in the Awash Valley, North eastern Ethiopia. Trans. R. Soc. Trop. Med. Hyg..

[bib0095] Gebre-Michael T., Malone J.B., Balkew M., Ali A., Berhe N., Hailu A., Herzi A.A. (2004). Mapping the potential distribution of Phlebotomus martini and P. orientalis (Diptera: Psychodidae), vectors of kala-azar in East Africa by use of geographic information systems. Acta Trop..

[bib0100] Gebre-Michael T., Balkew M., Alamirew T., Gudeta N., Reta M. (2007). Preliminary entomological observations in a highland area of Amhara Region, northern Ethiopia, with epidemic visceral leishmaniasis. Ann. Trop. Med. Parasitol..

[bib0105] Gebre-Michael T., Balkew M., Berhe N., Hailu A., Mekonnen Y. (2010). Further studies on the phlebotomine sand flies of the kala-azar endemic lowlands of Humera- Metema (North-West Ethiopia) with observations on their natural blood meal sources. Parasite Vectors.

[bib0110] Gebresilassie A., Kirstein O.D., Yared S., Aklilu E., Moncaz A., Tekie H., Balkew M., Warburg A., Hailu A., Gebre-Michael T. (2015). Species composition of phlebotomine sand flies and bionomics of *Phlebotomus orientalis* (Diptera: psychodidae) in an endemic focus of visceral leishmaniasis in Tahtay Adiyabo district, Northern Ethiopia. Parasites Vectors.

[bib0115] Gemetchu T., Zerihune A., Assefa G., Lemma A. (1975). Observations on the sand fly (Phlebotomidae) fauna of Setit-Humera (Northwestern Ethiopia). Ethiop. Med. J..

[bib0120] Gemetchu T. (1983). The distribution of sand flies (Diptera, Psychodidae, Phlebotominae) in north-west Ethiopia. Sinet Ethiop. J. Sci..

[bib0125] Ghosh K.N., Mukhopadhyay J.M., Guzman H., Tesh R.B., Munstermann L.E. (1999). Interspecific hybridization of genetic variability of *Phlebotomus* sand flies. Med. Vet. Entomol..

[bib0130] Guernaoui S., Pesson B., Boumezzough A., Pichon G. (2005). Distribution of phlebotomine sandflies, of the subgenus *Larroussius*, in Morocco. Med. Vet. Entomol..

[bib0135] Guernaoui S., Boussaa S., Pesson B., Boumezzough A. (2006). Nocturnal activity of phlebotomine sand flies (Diptera Pyschodidae) in a cutaneous leishmaniasis focus in Chichaoua, Morroco. Parasitol. Res..

[bib0140] Hailu A., Balkew M., Berhie N., Meredith S.E.O., Gemetchu T. (1995). Is *Phlebotomus* (*Larroussius*) *orientalis* a vector of visceral leishmaniasis in South-west Ethiopia?. Acta Trop..

[bib0145] Hailu A., Gebre-Michael T., Berhe N., Balkew M., Kloos H., Berhane Y., Hailemariam Addis Ababa D. (2006). Leishmaniasis. Epidemiology and Ecology of Health and Disease in Ethiopia.

[bib0150] Hamilton J.A. (2005). Species diversity or biodiversity?. J. Environ. Manag..

[bib0155] Herrero M., Orfanos G., Argaw D., Mulugeta A., Aparicio P., Parreno F., Bernal O., Rubens D., Pedraza J., Lima A.M., Flebaud L., Palma P., Bashaye S., Alvar J., Bern C. (2009). Natural history of a visceral leishmaniasis outbreak in highland Ethiopia. Am. J. Trop. Med. Hyg..

[bib0160] Hoogstraal H., Heyneman D. (1969). Leishmaniasis in the Sudan Republic 30. Final epidemiologic report. Am. J. Trop. Med. Hyg..

[bib0165] Lane R.P., Fritz G. (1986). The differentiation of the leishmaniasis Phlebotomus papatasi from the suspected P. bergeroti (Diptera: phlebotominae). Syst. Entomol..

[bib0170] Lane R.P., Lane R.P., Crosskey R.W. (1993). Sand flies (Phlebotominae). Medical Insects and Arachnids.

[bib0175] Lewis D.J. (1982). A taxonomic review of the genus *Phlebotomus* (Diptera: Psychodidae). Bull. Br. Mus. Nat. Hist..

[bib0180] Marlet M.V.L., Sang D.K., Ritmeijer K., Muga R.O., Onsongo J., Davidson R.N. (2003). Emergence or re-emergence of visceral leishmaniasis in areas of Somalia, north-eastern Kenya, and south-eastern Ethiopia in 2000–01. Trans. R. Soc. Trop. Med. Hyg..

[bib0185] Minter D.M. (1964). Seasonal changes in populations of phlebotomine sand flies (Diptera: Psychodidae) in Kenya. Bull. Entomol. Res..

[bib0190] Ngure P.K., Kimutai A., Ng’ang’ Z.W., Rukunga G., Tonui W.K. (2009). A review of leishmaniasis in Eastern Africa. JNMU.

[bib0195] Quate L.W. (1964). *Phlebotomus* sand flies of the Paloich Area in the Sudan (Diptera, Psychodidae). J. Med. Entomol..

[bib0200] Rohr J.R., Dobson A.P., Johnson P.T., Kilpatrick A.M., Paull S.H., Raffel T.R., Ruiz-Moreno D., Thomas M.B. (2011). Frontiers in climate change-disease research. Trends Ecol. Evol..

[bib0205] Simsek F.M., Alten B., Caglar S.S., Ozbel Y., Aytekin A.M., Kaynas S., Belen A., Kasap O.E., Yaman M., Rastgeldi S. (2007). Distribution and altitudinal structuring of phlebotomine sand flies (Diptera: Psychodidae) in southern Anatolia, Turkey: their relation to human cutaneous leishmaniasis. J. Vector Ecol..

[bib0210] Thomson M.C., Elnaiem D.A., Ashford R.W., Connor S.J. (1999). Towards a kala azar risk map for Sudan: mapping the potential distribution of *Phlebotomus orientalis* using digital data of environmental variables. Trop. Med. Int. Health.

[bib0215] Tsegaw T., Gadisa E., Seid A., Abera A., Teshome A., Mulugeta A., Herrero M., Argaw D., Jorge A., Aseffa A. (2013). Identification of environmental parameters and risk mapping of visceral leishmaniasis in Ethiopia by using geographical information systems and a statistical approach. Geospat. Health.

[bib0220] WHO (2010). Control of the Leishmaniasis: Report of a Meeting of the WHO Expert Committee on the Control of Leishmaniases.

[bib0225] Wilkins H.A. (1972). Studies on leishmaniasis in Ethiopia VI: Incidence rates of cutaneous leishmaniasis at Meta Abo. Ann. Trop. Med. Parasitol..

[bib0230] Yared S., Deribe K., Gebreselassie A., Lemma W., Akililu E., Kirstein O.D., Balkew M., Warburg A., Gebre-Michael T., Hailu A. (2014). Risk factors of visceral leishmaniasis: a case control study in north-western Ethiopia. Parasite Vectors.

[bib0235] Yared S., Gebresilassie A., Akililu E., Balkew M., Warburg A., Hailu A., Gebre-Michael T. (2017). Habitat preference and seasonal dynamics of Phlebotomus orientalis in urban and semi-urban areas of kala-azar endemic district of Kafta Humera, northwest Ethiopia. Acta Trop..

